# Supplementary material for: The interactome of intact mitochondria by cross-linking mass spectrometry provides evidence for coexisting respiratory supercomplexes
Source: Mol Cell Proteomics. 2017 Dec 8;17(2):216–32. doi: 10.1074/mcp.RA117.000470 (PMC5795388; doi:10.1074/mcp.RA117.000470)
Supplement: Supplemental Data [file supp_RA117.000470_133922_0_supp_23978_fzffwf.zip › spectra_annotation/mito_spectra_annotation/83-1-9-1-10-1.pdf]

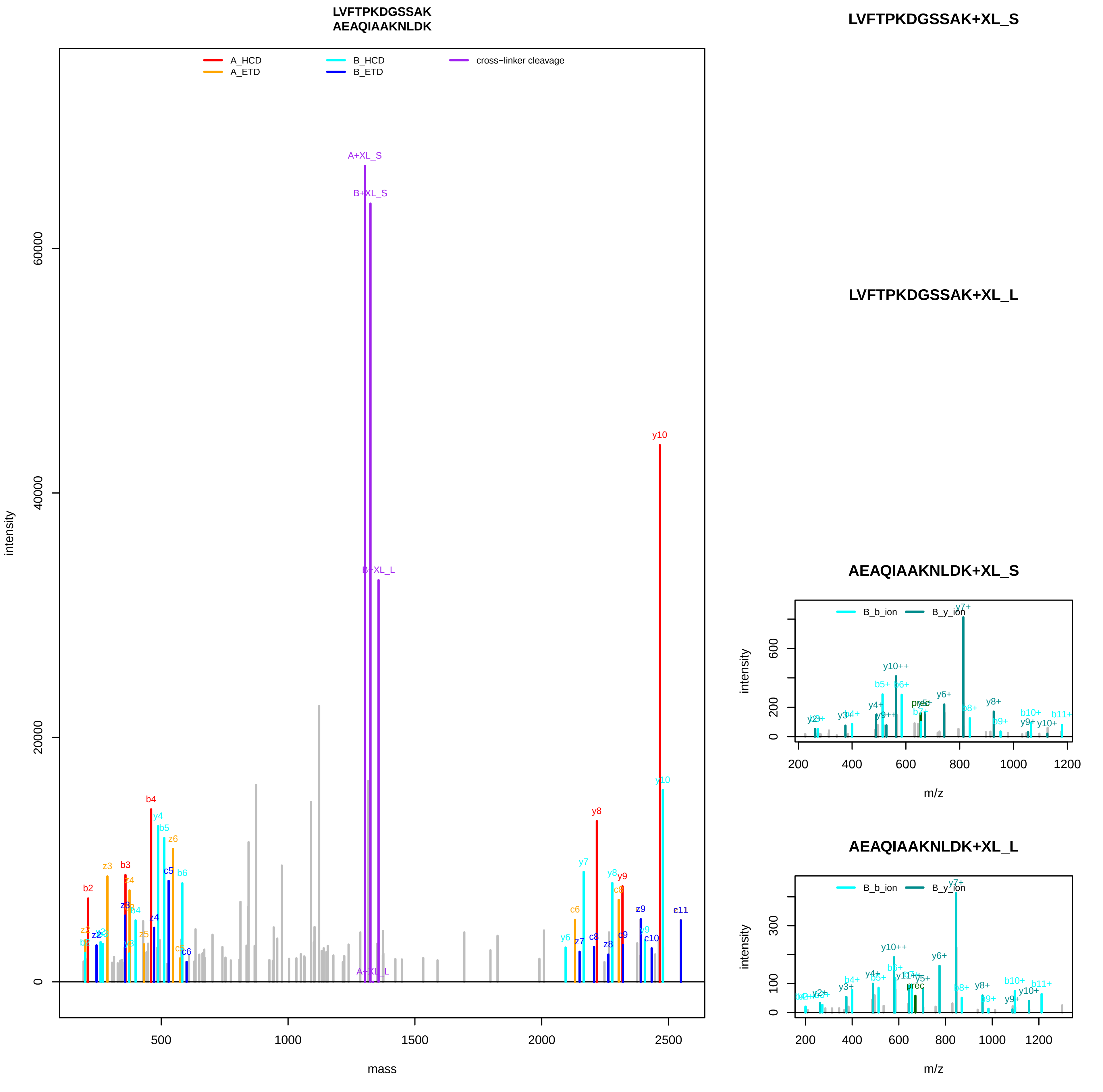
LVFTPKDGSSAK
AEAQIAAKNLDK
A_HCD
A_ETD
B_HCD
B_ETD
cross−linker cleavage
A+XL_S
B+XL_S
B+XL_L
A+XL_L
y10
y8
y9
c8
c6
y7
y6
z7
z8
c9
z9
y9
c10
c11
b2
b3
b4
y4
b5
z6
c5
b6
z3
z4
z2
z5
c6
intensity
mass
0
20000
40000
60000
500
1000
1500
2000
2500
LVFTPKDGSSAK+XL_S
LVFTPKDGSSAK+XL_L
AEAQIAAKNLDK+XL_S
B_b_ion
B_y_ion
y7+
y10++
b5+
b6+
y4+
y6+
y8+
b8+
b10+
b11+
y10+
prec
intensity
m/z
0
200
400
600
800
1000
1200
AEAQIAAKNLDK+XL_L
B_b_ion
B_y_ion
y7+
y10++
y6+
b4+
y4+
b5+
y5+
y11++
y8+
b10+
b11+
y10+
y9+
b8+
y2+
y3+
prec
intensity
m/z
0
100
300
200
400
600
800
1000
1200
